# Extramedullary plasmacytoma of the cricoid cartilage progressing to multiple myeloma: A case report

**DOI:** 10.3892/ol.2015.2936

**Published:** 2015-02-05

**Authors:** MIN WANG, JINTAO DU, JIAN ZOU, SHIXI LIU

**Affiliations:** Department of Otorhinolaryngology-Head and Neck Surgery, West China Hospital, Sichuan University, Chengdu, Sichuan 610041, P.R. China

**Keywords:** solitary plasmacytoma, multiple myeloma, larynx, cricoid cartilage

## Abstract

Extramedullary plasmacytoma (EMP) is an uncommon tumor of the plasma cells, and EMP of the cricoid cartilage is extremely rare The present study reports the rare case of a 43-year-old male complaining of hoarseness and dyspnea for over a year. Computed tomography imaging of the neck revealed an occupying lesion in the cricoid cartilage, and a diagnosis of EMP was subsequently confirmed by histopathological evaluation. The patient underwent surgery, but refused radiotherapy against medical advice. One year later, the patient was diagnosed with a recurrence of EMP via pathological examination and underwent a second surgery, however, radiotherapy was refused. After a further two years and four months, the patient presented with lumps in the neck, and was subsequently diagnosed with multiple myeloma. The patient received systematic chemotherapy in the hematology department; the patient’s general condition stabilized, and no further anemia, bone pain, infection or other discomfort was experienced. Solitary plasmacytoma of the cricoid cartilage is a rare lesion and compliance with radiotherapy following surgery may provide significant benefit in the treatment of this disease.

## Introduction

Extramedullary plasmacytoma (EMP) of the larynx is an uncommon neoplasm of plasma cells, which is extremely rarely observed in the cricoid cartilage. A previous study reported that 80–90% EMP cases occur in the head and neck (1), but only three case reports have described EMP occurring in the cricoid cartilage (2–4). The typical treatments for this disease are irradiation, surgery, or irradiation combined with surgery in certain locations such as the head and neck, with a 50–80% survival rate after 10 years (5). However, the most important factor that influences the prognosis of the disease is the development to myeloma (6). The current study reports one case of EMP, subsequently progressing to multiple myeloma. The treatment and collection of information for this study was approved by the ethics committee of West China Hospital, Sichuan University (Chengdu, China). Written informed consent was obtained from the patient.

## Case report

**Figure 1:**
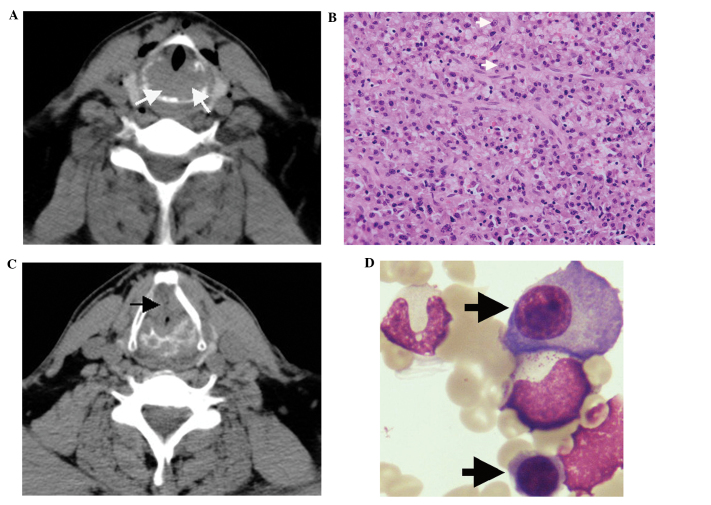
(A) CT imaging of the neck revealed a space-occupying lesion of cricoid cartilage on initial admission (white arrow) and (B) histopathological examination showed a mass of plasmocytes (white arrow). (C) CT imaging revealed thickened cricoid cartilage and bone destruction (black arrow) at the second admission. (D) Bone marrow smear from the posterior superior iliac spine showed active proliferation of plasmocytes (black arrows) over two years following the second surgery. CT, computed tomography.

A 43-year-old male was admitted to the Department of Otorhinolaryngology of West China Hospital on June 2, 2009, complaining of hoarseness lasting for over two years and dyspnea for over one year, which had intensified for a week. An emergency tracheostomy was performed under local anesthesia. Electronic laryngoscopy revealed swelling of the arytenoid, vocal cord, laryngeal ventricle and ventricular band, and also indicated limited movement of the right vocal cord and subglottic stenosis. However, no neoplasm was observed in the laryngeal cavity or main bronchus. Computed tomography (CT) imaging of the neck revealed a space-occupying lesion of the cricoid cartilage (Fig. 1A), with enlargement of the left lymph nodes. Three days after hospitalization, the patient underwent partial laryngectomy, reconstruction of laryngeal function, and left cervical lymphadenectomy.

Surgery revealed erosion of the posterior and lateral cricoid cartilage (fish meat-like tissue was observed), and two swollen lymph nodes in the second left cervical area. The invaded tissue was completely excised, and the laryngeal cavity was reconstructed. Histopathological analysis revealed laryngeal plasmacytoma (Fig. 1B) without lymph node metastasis. The patient was diagnosed with solitary EMP of the larynx following positron emission tomography (PET)-CT and immunofixation electrophoresis. PET-CT revealed no tumors, cancer or lytic bone metastases and immunofixation electrophoresis did not reveal monoclonal immunoglobulin. Against medical advice, the patient refused radiotherapy suggested by the Department of Oncology, for financial reasons.

One year later, the patient was readmitted after complaining of dysphonia, and electronic laryngoscopy revealed the presence of extensive adhesion and stenosis in the subglottic tissue. CT imaging of the neck showed thickening of the left wall of the subglottic area, and the cricoid cartilage was broken (Fig. 1C). Tumor recurrence was suspected, and therefore a second surgery was performed.

During surgery, an irregular callus was identified in the middle of the cricoid cartilage, which was causing airway stenosis. The left side of the cricoid cartilage had been destroyed by the tumor. Complete resection of the tumor tissue and the left cricoid cartilage was performed in order to expand the airway. On histopathological evaluation, the postoperative diagnosis was determined to be recurrence of solitary plasmacytoma of the larynx. As previously, against medical advice, the patient refused to undergo radiotherapy following surgery. Two years and four months after the second surgery, the patient presented with osseous masses in the bilateral clavicle, right cheekbone, and ribs, and was subsequently diagnosed with multiple myeloma based on whole body radiography, a bone marrow smear showing that plasmocytes accounted for 15% of the total cells(Fig. 1D), significantly active proliferation shown by the bone marrow biopsy and immunofixation electrophoresis confirming the type of λ light chain. The patient received five systematic chemotherapy, each cycle lasting one month, in the hematology department, which consisted of the MPT regimen: Melphalan (4 mg, three times a day, days 1–4), prednisone (45 mg, three times a day, days 1–4) and thalidomide (100 mg, once a day, days 1–28). The patient’s general condition stabilized, and no further anemia, bone pain, infection or other discomfort was experienced. The patient remains alive after 4 years of follow-up, and follow-up will continue to screen for recurrence.

## Discussion

Solitary plasmacytoma is a rare malignant plasma cell tumor that is caused by the monoclonal proliferation of plasma cells, and may be classified as EMP or solitary plasmacytoma of the bone. According to the National Comprehensive Cancer Network Guidelines for multiple myeloma (version 1.2013), the patient in the current case was diagnosed with EMP. EMP is typically observed in males aged from 40–70 years (7) and accounts for approximately 3% of plasma cell neoplasms, and <1% of head and neck tumors (8). It is three times more common in males than in females (9).

The diagnosis of solitary plasmacytoma of the cricoid cartilage is predominantly determined using histopathology, following the exclusion of multiple myeloma (10). The preferred treatment for EMP is radiotherapy at 45 Gy and/or surgical intervention for the involved region (9). EMP is highly radiosensitive, with an 80–100% local control rate following radical radiotherapy. However, chemotherapy is preferred for cases involving whole-body dissemination (10). EMP prognosis is associated with the extent of differentiation; tumors with lower differentiation are more likely to develop into multiple myeloma (11). Hematoxylin and eosin staining revealed a low differentiation of tumor cells acquired from our patient, which was consistent with previous studies (11,12).

Although radiotherapy and surgery are both viable treatment options for solitary plasmacytoma, complete surgical resection of the tumors may not be possible in cases involving the primary cricoid cartilage due to the necessity to preserve larynx function. In such cases, patients should receive radiotherapy following surgery (13). Clinicians must also be alert to signs of progression to multiple myeloma, such as anemia, bone pain and renal insufficiency, particularly in cases of relapse.
